# Modelling non‐linear psychological processes: Reviewing and evaluating non‐parametric approaches and their applicability to intensive longitudinal data

**DOI:** 10.1111/bmsp.12397

**Published:** 2025-05-30

**Authors:** Jan I. Failenschmid, Leonie V. D. E. Vogelsmeier, Joris Mulder, Joran Jongerling

**Affiliations:** ^1^ Tilburg University Tilburg The Netherlands

**Keywords:** Gaussian process, generalized additive model, intensive longitudinal data, local polynomial regression, non‐linearity, non‐parametric regression

## Abstract

Psychological concepts are increasingly understood as complex dynamic systems that change over time. To study these complex systems, researchers are increasingly gathering intensive longitudinal data (ILD), revealing non‐linear phenomena such as asymptotic growth, mean‐level switching, and regulatory oscillations. However, psychological researchers currently lack advanced statistical methods that are flexible enough to capture these non‐linear processes accurately, which hinders theory development. While methods such as local polynomial regression, Gaussian processes and generalized additive models (GAMs) exist outside of psychology, they are rarely applied within the field because they have not yet been reviewed accessibly and evaluated within the context of ILD. To address this important gap, this article introduces these three methods for an applied psychological audience. We further conducted a simulation study, which demonstrates that all three methods infer non‐linear processes that have been found in ILD more accurately than polynomial regression. Particularly, GAMs closely captured the underlying processes, performing almost as well as the data‐generating parametric models. Finally, we illustrate how GAMs can be applied to explore idiographic processes and identify potential phenomena in ILD. This comprehensive analysis empowers psychological researchers to model non‐linear processes accurately and select a method that aligns with their data and research goals.

## 1 INTRODUCTION

Psychological constructs are increasingly understood as components of complex dynamic systems (Nesselroade & Ram, 2004; Wang et al., 2012). This perspective emphasizes that these constructs fluctuate over time and within individuals. To study these variations and the underlying processes, researchers are increasingly collecting intensive longitudinal data (ILD) using ecological momentary assessment (EMA), experience sampling or similar methods (Fritz et al., 2023). In these studies, one or more individuals are assessed at a high frequency (multiple times per day) using brief questionnaires or passive measurement devices. These rich data allow researchers to examine complex temporal variations in the underlying psychological variables within an ecologically valid context and to explain them through (between‐person differences) in within‐person processes.

**FIGURE 1:**
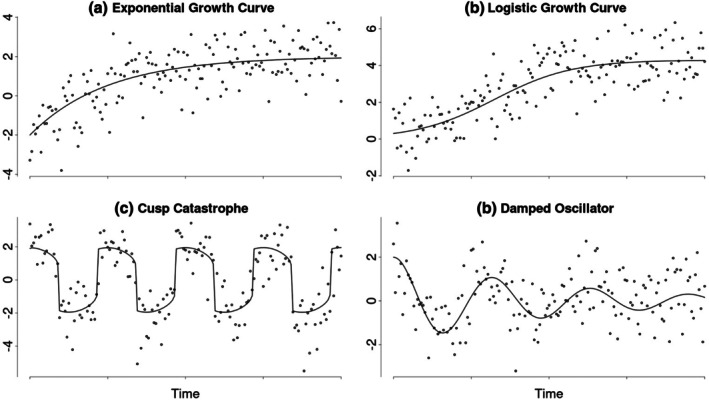
Examples of non‐linear processes demonstrated to occur in psychological time series. This figure shows four demonstrated psychological non‐linear processes. These processes were modelled using common deterministic or ordinary differential equation models. Panels (a) and (b) show exponential and logistic growth curves, respectively. Panel (c) shows a cusp catastrophe model. Lastly, panel (d) shows a damped oscillator.

Through ILD studies, many non‐linear psychological phenomena and processes have been discovered in recent years, which either show monotonically increasing or decreasing non‐linear trajectories or non‐monotonic gradual or even abrupt change. Clear examples of gradual monotonically increasing processes are the learning and growth curves observed in intellectual and cognitive development (Kunnen, 2012; McArdle et al., 2002). In these cases, an individual's latent ability increases over time, following an intricate non‐linear trajectory from a (person‐specific) starting point towards a (person‐specific) asymptote, which reflects the individual's maximum ability. Additional examples of asymptotic growth over time spans that are typically studied with ILD include motor skill development (Newell et al., 2001) and second language acquisition (De Bot et al., 2007). Figure 1 shows common deterministic^1^ model choices for these kinds of processes in the form of an exponential growth function (a) and a logistic growth function (b). Both of these models describe asymptotic trends towards a person mean. However, whereas the logistic model only describes asymptotic growth from zero, the exponential model can also capture different starting values and asymptotic decay. Additionally, the exponential model has its largest rate of change in the beginning, while the logistic model starts off changing slowly and has its largest rate of change when reaching the midpoint between zero and the asymptote.

A common example of non‐monotonic abrupt change is switching between seemingly distinct states that differ, for instance, in their means. This occurs, for example, during the sudden perception of cognitive flow, where individuals abruptly switch from a ‘normal’ state to a flow state and back (Ceja & Navarro, 2012). Another example of this is alcohol use relapse, where patients suddenly switch from an abstinent state to a relapsed state (Witkiewitz & Marlatt, 2007). This sudden switching behavior has been modelled using a cusp catastrophe model (Chow et al., 2015; van der Maas et al., 2003). This dynamic model has been exemplified in Figure 1c. One of the behaviors that the cusp model is well‐known for is that it can suddenly switch between two seemingly distinct mean levels, when one of its parameters is varied.

As a final example, we consider non‐monotonic gradual change. This can be found, for example, in (self‐) regulatory systems, which maintain a desired state by counteracting external perturbations. In these systems, the degree of regulation often depends on the distance between the current and the desired states. The common lag‐one autoregressive model describes such a system in which the regulation strength depends linearly on this distance, resulting in an exponential return to the person mean. However, this relationship may also be non‐linear, such that the degree of regulation changes disproportionately with larger distances. One example of such a possible (self‐) regulatory system is emotion regulation, which has been modelled using a damped oscillator model (Chow et al., 2005). This model is exemplified in Figure 1d. The damped oscillator model describes decreasing oscillations around a mean and it was originially devised to describe the oscillations of a pendulum returning back to rest.

Although initial evidence for non‐linearity in psychological research exists, theories about the nature and form of non‐linear psychological processes remain scarce (Tan et al., 2011). Frequently, psychological theories are too general to result in specific hypotheses (Oberauer & Lewandowsky, 2019), such as, about the specific form of non‐linear dynamics. ILD studies can potentially help to refine such theories by providing a nuanced understanding of how psychological variables interact over time. These refined theories could, for instance, take the form of parametric dynamic models, such as differential equation (Boker, 2012) or state‐space models (Durbin & Koopman, 2012) that describe how a given process changes over time. However, in order to develop these more specific theories (about the form of non‐linear change), it is first necessary to empirically uncover, observe, and study phenomena such as the mentioned state switching or regulatory oscillations in ILD and determine if they generalize beyond individual data sets and contexts. Formal theories about the underlying process should then be able to explain these phenomena and different candidate theories can be compared based on their success in doing so (Borsboom et al., 2021). While the study of non‐linear phenomena in ILD is receiving increasingly more attention in psychology and different statistical techniques are developed to explore these phenomena (Cui et al., 2023; Humberg et al., 2024), researchers are currently still limited in their ability to infer non‐linear phenomena from ILD. One reason for this is that advanced statistical methods that are flexible enough to adequately capture and explore these processes are currently not well established in psychological research, which hinders the development and evaluation of guiding theories.

Due to adequate statistical methods not being commonly available, non‐linear trends in psychology are often addressed through polynomial regression (Jebb et al., 2015). Polynomial regression uses higher‐order terms (e.g. squared or cubed time, in addition to a linear effect of time) as predictors in a standard multiple linear regression model. While effective for relatively simple non‐linear relationships, particularly those that can be represented as polynomials, this method has significant limitations and likely leads to invalid results when applied to more complex processes, such as mean switching or (self‐) regulatory systems (e.g. Figure 1c,d).

When fitting models with many degrees of freedom, such as a polynomial regression with many higher‐order terms, the risk of underfitting or overfitting arises quickly. Underfitting occurs when the model is too simple or inflexible to accurately capture the underlying process, whereas overfitting occurs when the model is overly flexible and adapts to random noise in the data, resulting in poor generalization and unreliable predictions for new samples. For complex processes, polynomial approximations often require many higher‐order terms to accurately capture the intricate trajectory. However, the large number of highly correlated parameters in these models can lead to model instability, which can cause convergence issues during estimation. This instability may force researchers to use simpler models, even though these might not be able to capture more complex processes accurately. Even when the polynomial degree is optimal, polynomial regression can result in overfitting at the boundaries of the process while underfitting the central regions, because polynomials are defined over the entire range of the data and (from quadratic terms onwards) get more wiggly further away from zero. This may also produce non‐sensical predictions, such as interpolating scores outside the valid range of the scale (Boyd & Xu, 2009; Harrell, 2001; Jianan et al., 2023; Magee, 1998).^2^

These limitations in the currently available methods underscore the need for more sophisticated and data‐driven statistical methods to study and explore non‐linear processes. Various such advanced statistical methods like local polynomial regression (Fan & Gijbels, 2018), Gaussian processes (Rasmussen & Williams, 2006) and generalized additive models (Wood, 2006) are available outside of psychology. However, these methods have rarely been applied in psychology because they have not been reviewed for an applied audience, nor have their assumptions and inference possibilities been evaluated in the context of ILD. As a result, it is difficult for psychological researchers to select the most suitable method for a specific context. Moreover, the ideal statistical method may depend on the characteristics of the underlying non‐linear process (which, as yet, are generally unknown). Especially, since the types of non‐linear processes for which the different methods were developed match the types of processes occurring in psychological research (e.g. the processes depicted in Figure 1) to varying degrees, particularly with regard to the assumed smoothness of the non‐linear process. This means that the method most suitable for the relatively smooth process in Figure 1 is not necessarily also the most suitable method for rougher changes.

To address this important gap, this article reviews three advanced non‐linear analysis methods, local polynomial regression, Gaussian processes and generalized additive models and evaluates their applicability to typical ILD scenarios (Section 2). The methods reviewed in this article are various semi‐ and non‐parametric regression techniques that were chosen for their ability to model non‐linear processes while accommodating different levels of prior knowledge. Our review specifically focuses on methods that provide a clear and transparent mechanism for inferring non‐linear processes and for preventing over‐ and underfitting the data. Further, we limited our selection to methods that are implemented in the open‐source software R (R Core Team, 2024), making them readily accessible for research applications. Moreover, we prioritized methods that are promising for application to psychological ILD, particularly in terms of sample size and measurement frequency requirements. We additionally compare how well each method recovers different non‐linear processes under common ILD conditions in a simulation study (Section 3). Lastly, we demonstrate how the best‐performing method can be applied to analyze an existing data set (Section 4). Note that, to introduce these methods accessibly and apply them under conditions where software implementations are available, this article focuses on the univariate single‐subject design. This aligns with the growing trend in psychology focusing on idiographic research (e.g., Hamaker, 2012; Klaassen et al., 2017; Molenaar, 2004). Furthermore, we demonstrate in the empirical example how estimates of idiographic processes can provide valuable insights into between‐person heterogeneity.

## 2 NON‐LINEAR ANALYSIS METHODS

In the following paragraphs, the three semi‐ and non‐parametric regression techniques will be introduced: local polynomial regression, Gaussian processes, and generalized additive models. These models make it possible for researchers to explore the shape of psychological processes in a data‐driven manner without making assumptions regarding the functional form of the process. Hence, they fall into the same niche in which polynomial regression is currently often applied. Uncovering the shape and behaviour of psychological processes is particularly relevant for theory building as it informs the features that possible candidate theories need to be able to capture and explain. For instance, if a process consistently displays decreasing oscillations or switches between different means, then theory‐driven parametric models can be developed and subsequently tested, which can capture these specific behaviours. Additionally, some of the methods introduced below can combine incomplete parametric models with flexible data‐driven components. This makes it possible to test partial theories while maintaining the necessary flexibility to accurately capture the underlying processes. Therefore, these methods are particularly well suited for iterative theory development. In the following we provide an introduction to the statistical theory underlying each method and show how each method can be implemented in practice.

### 2.1 Local polynomial regression

#### 2.1.1 Statistical theory

**FIGURE 2:**
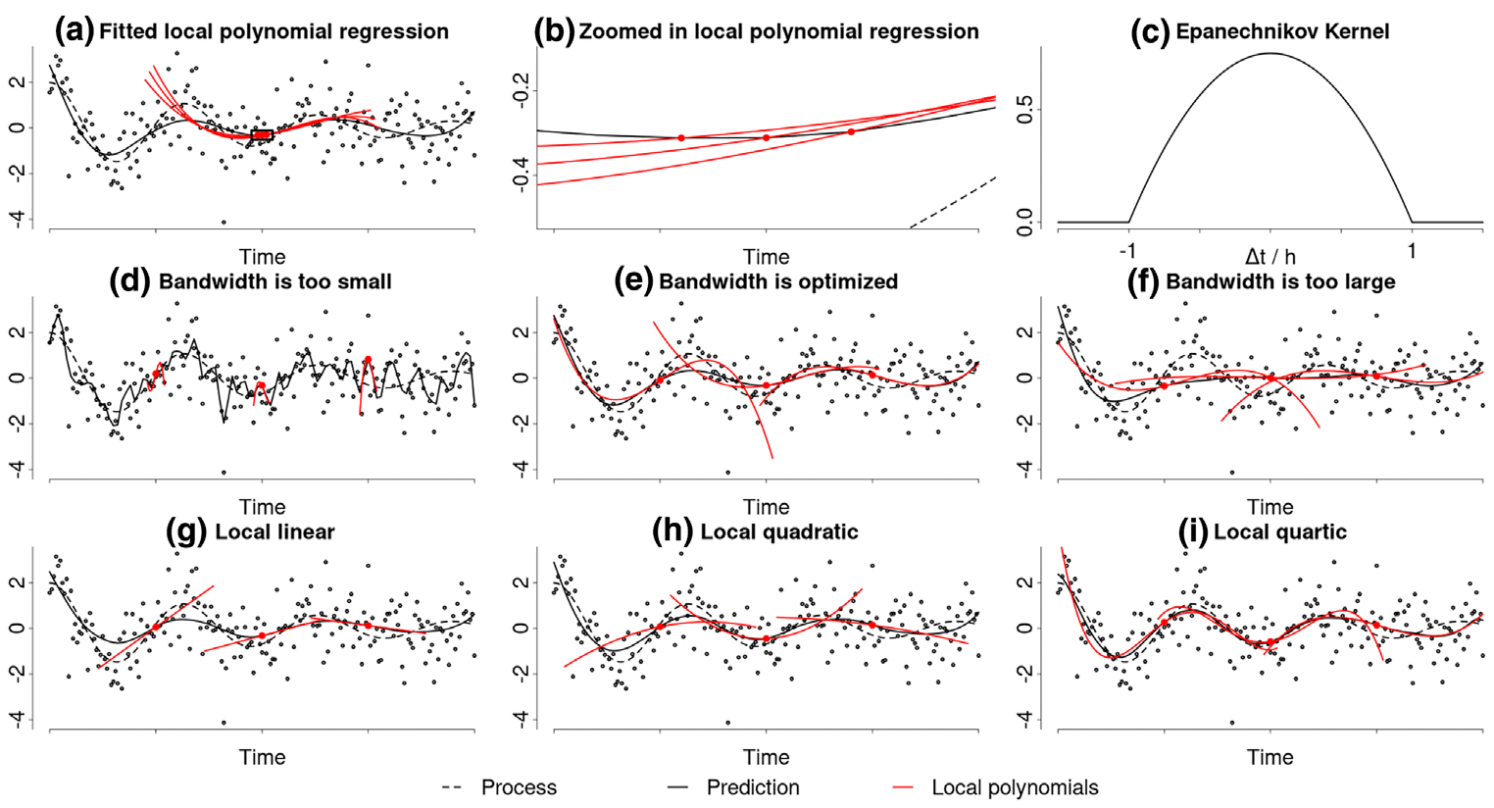
Demonstration of a local polynomial regression. This figure shows how LPR (solid black) estimates the underlying process (dotted black). Panel (a) shows three of the 100 local cubic regressions used to construct the overall estimate of the process (red). Panel (b) provides a zoomed‐in view of a specific section of panel (a), illustrating that the estimated process is composed of the intercepts of the three local cubic regressions. Panel (c) depicts the Epanechnikov kernel that was used in the LPR depicted in panel (a). Panels (d‐f) illustrate the impact of varying the bandwidth of a local cubic regression. Panel (d) highlights overfitting resulting from a bandwidth that is too narrow, panel (e) shows an optimized bandwidth and panel (f) depicts underfitting caused by a bandwidth that is too wide. Lastly, panels (g‐i) display optimized local linear (g), quadratic (h) and quartic (i) regressions, showing the effect of varying the polynomial degree.

The first technique we are reviewing is called local polynomial regression (LPR). Similarly to regular polynomial regression, LPR approximates the process using polynomial basis functions (e.g. squared or cubed time). However, instead of using one large polynomial function to approximate the entire process, LPR estimates smaller, local polynomials at every point in time. These local polynomials are then combined into a single non‐linear function over the entire set of observations (Fan & Gijbels, 1995a, 2018; Ruppert & Wand, 1994). This technique is a generalization of the rolling average for time series smoothing and relies on the same underlying principle. The idea behind the rolling average is that the value that the process takes at a particular point in time can be approximated by averaging the observations that are closer in time. This approach allows researchers to estimate the process's value at any chosen set of time points. Subsequently, these distinct estimates can be combined to form an overall picture of the non‐linear process. LPR generalizes this principle by using more flexible polynomial regressions instead of an average to approximate the process pointwise. Figure 2a shows the estimated LPR (solid black line) for the damped oscillator example process (dotted black line) introduced in Figure 1d. Three of the 100 local cubic polynomials that were used to estimate this process are shown in red. Figure 2b zooms in to illustrate how each local cubic regression estimates the process value at a single time point, which are subsequently connected into an overall estimate of the process.

Specifically, to predict the value of the univariate process f at an arbitrary time point t* based on the set of observations $y_{t}$ at the time points t, the LPR starts by centring the data around the time point t* (by shifting the data along the time axis so that the chosen time point is at zero). Afterwards, a low order polynomial regression is fit to the data around t*.

(1) $$\begin{array}{ll} y_{t} & =f(t)+\epsilon_{t}\; \end{array}$$

(2) $$\begin{array}{ll} \text{X}(t^{*}) & =\left[\begin{array}{cccc} 1 & {\left(t_{1}-t^{*}\right)}^{1} & \text{…} & {\left(t_{1}-t^{*}\right)}^{p}\\ \vdots & \vdots & \ddots & \vdots \\ 1 & {\left(t_{n}-t^{*}\right)}^{1} & \text{…} & {\left(t_{n}-t^{*}\right)}^{p} \end{array}\right]\; \end{array}$$

This polynomial regression uses the predictor matrix X, in which each column corresponds to a polynomial transformations of time centred around $t^{*}$ (i.e. the first column is filled with ones, the second column contains the centred time points, the third column contains the centred time points squared and so on, up to the chosen degree p of the polynomial). The degree p of the polynomial regression reflects an assumption about how smooth the underlying process is. Specifically, for a first‐degree LPR, the process should not exhibit any corners, discontinuities or vertical sections. This ensures that the process's rate of change (i.e. first derivative), approximated by the first‐order polynomial term, is well‐behaved. Higher‐order local polynomials require this smoothness for increasingly complex rates of change. For instance, a second‐degree LPR requires that the rate of change itself is smooth, which ensures that its rate of change is well‐behaved. This property should hold for all p rates of change of a process when using an LPR with p degrees.^3^

Since this polynomial approximation is more accurate for data points closer in time, an additional weighting function is introduced in the polynomial regression, which assigns deminishing weights to points that are further away from $t^{*}$.

(3) $$\begin{array}{cc} \text{W}(t^{*}) & =\left[\begin{array}{ccc} w_{1,1}(t^{*}) & & \\ & \ddots & \\ & & w_{n,n}(t^{*}) \end{array}\right] \end{array}$$

(4) $$\begin{array}{ll} w{\left(t^{*}\right)}_{i,i} & =k_{\mathrm{LPR}}(\frac{\left|t_{i}-t^{*}\right|}{h})\; \end{array}$$

The exact weights for each datum are determined by a kernel function $k_{\mathrm{LPR}}$. Common choices for this kernel function are the Gaussian, the Epanechnikov (Figure 2c) or the uniform kernel. The Gaussian and Epanechnikov kernels assign higher weights to data points in closer proximity to the point of interest $t^{*}$; however, while the Gaussian kernel assigns small weights to all distant data points, the Epanechnikov kernel assigns zero weights beyond a certain distance. The uniform kernel assigns equal weights to all data points within a certain distance and zero weights beyond this distance. The kernel function additionally includes a bandwidth parameter h, which controls the width of the kernel and regulates the rate at which the weights decrease with increasing distance.

Finally, the value that the LPR predicts for $t^{*}$ is given by the intercept of the fitted locally weighted polynomial around $t^{*}$. For such a weighted polynomial regression the coefficient vector $\hat{\beta }$, whose first element is the intercept, can be obtained through the normal equation in Equation (5).

(5) $$\begin{array}{ll} \hat{\beta }(t^{*}) & ={\left(\text{X}{\left(t^{*}\right)}^{T}\text{W}(t^{*})\text{X}(t^{*})\right)}^{-1}\text{X}{\left(t^{*}\right)}^{T}\text{W}(t^{*})\text{y}\; \end{array}$$

(6) $$\begin{array}{ll} \hat{f}(t^{*}) & ={\hat{\beta }}_{1}(t^{*})\; \end{array}$$

This procedure is repeated for all time points of interest and the estimated LPR values are subsequently connected into an overall estimate of the nonlinear process. As it is theoretically possible to repeat this process at infinitely many time points, LPR is a non‐parametric technique.

#### 2.1.2 Practical implementation

When fitting an LPR, researchers need to make three decisions concerning the kernel function, the bandwidth of the kernel and the degree of the local polynomials. As described in the previous section, the kernel function determines how the weights for the data points are distributed during the estimation of the local polynomials. Common choices for the kernel function include the Gaussian, the Epanechnikov or the uniform kernel. Because the Epanechnikov kernel has been shown to be optimal in many situations (Fan et al., 1997), it constitutes a good default choice if there are no strong preferences for other kernels. In practice, different kernel functions rarely result in strongly differing estimates. Nevertheless, it is advisable to conduct sensitivity analyses to assess whether any conclusions depend (strongly) on the chosen kernel.

The bandwidth parameter, which as previously described determines the width of the kernel function, effectively controls the wiggliness of the estimated process. A bandwidth that is too small results in an LPR that is too flexible and overfits the data (Figure 2d). Conversely, a bandwidth that is too large results in an LPR that is not flexible enough and underfits the data (Figure 2f). Therefore, it is critical to the performance of the LPR to find an optimal bandwidth that strikes a balance between being flexible enough to adequately capture the underlying process and not overfitting the data (Figure 2e). Several methods are available to find the optimal bandwidth by optimizing a data‐dependent criterion function, such as the cross‐validation error or the mean integrated squared error (Debruyne et al., 2008; Köhler et al., 2014). This optimization criterion may be selected based on specific research objectives. For instance, optimizing the cross‐validation error may be most attractive if the primary research interest is out of sample prediction. However, most standard software packages offer automated default procedures to find the optimal bandwidth, which is especially attractive for researchers new to LPR. Note that the fact that the LPR uses a single value for the bandwidth parameter implies that the method assumes that the underlying process has constant wiggliness (with respect to the degree of the local polynomials). This constant wiggliness assumption can be relaxed by using a time‐varying bandwidth (Fan & Gijbels, 1995b) or a time‐varying polynomial degree (Fan & Gijbels, 1995a), but these extensions are beyond the scope of this paper.

Lastly, the degree of the local polynomial must be chosen. Beyond reflecting an assumption about the smoothness of the process, higher‐degree polynomials offer greater flexibility to approximate the process using a single bandwidth. This can be seen in Figure 2, in which the local linear (g) and local quadratic (f) regressions are more rigid in approximating the process than the local cubic (a) or quartic (i) regressions, even though all models use an optimized bandwidth. It is important to note that unless the underlying process follows a polynomial trajectory of at most the same degree as the LPR, the approximation with local polynomials will introduce some bias. For example, while a process with a quadratic trajectory can be accurately inferred at any point by a local quadratic regression (or any higher‐order LPR), a process following an exponential trajectory cannot be perfectly captured by any LPR with a finite degree. Instead, there will be a small bias in the estimate, which decreases with higher polynomial degrees. This bias also affects the standard errors and confidence intervals estimated by the LPR. In standard practice, these confidence intervals are estimated around a biased point estimate, which can lead to non‐nominal coverage probabilities. While higher‐degree LPRs reduce bias, they also increase the estimator variance when transitioning from an odd to an even degree, leading to a bias‐variance trade‐off (Ruppert & Wand, 1994). As a result, the polynomial degree is typically chosen to be low and odd, with local linear and cubic regressions being the most commonly used and frequently result in sufficiently small biases. Additionally, there are also methods available to correct for this bias for both the point estimates and standard errors (Calonico et al., 2019).

An implementation of LPR can be found in the nprobust R package (Calonico et al., 2019). The LPR in Figure 1a can be fit using:

lprobust(y, time, eval = time, p = 3, kernel = 'epa', bwselect = 'imse‐dpi', bwcheck = 0)

Here we set the polynomial degree to three, chose an Epanechnikov kernel and specify that the bandwidth should be optimized by minimizing the integrated mean squared error. The LPR implemented in nprobust also automatically corrects for the possible bias introduced by the finite polynomial degree. The last argument is used to suppress an automated correction of the bandwidth towards the beginning and end of the process. This code returns bias‐corrected pointwise estimates of the process at the chosen evaluation points eval, which are depicted in Figure 2a, as well as robust standard errors. Additionally, it provides the optimized bandwidth parameter, which can be interpreted as an estimate of the wiggliness of the process with respect to the chosen polynomial degree.

### 2.2 Gaussian process regression

#### 2.2.1 Statistical theory

The second non‐parametric technique is Gaussian process (GP) regression, a Bayesian approach that directly defines a probability distribution over an entire family of non‐linear functions, which is flexible enough to capture many complex processes effectively (Betancourt, 2020; Rasmussen & Williams, 2006; Roberts et al., 2013). Unlike regular probability distributions (e.g. normal distributions) that specify the likelihood of single values, GPs determine how likely entire (non‐linear) functions are. A GP is defined indirectly, such that if the functions it describes are evaluated at any finite set of time points, the resulting sample of function values will follow a multivariate normal distribution.

In a Bayesian framework, one can use a GP to define a prior distribution for the latent process as f∼GP. Following Bayes' theorem, this prior is then combined with an appropriate likelihood for the observed data to obtain a posterior distribution for the latent process given the observed data:

(7) P(f|y)∝P(y|f)P(f)

**FIGURE 3:**
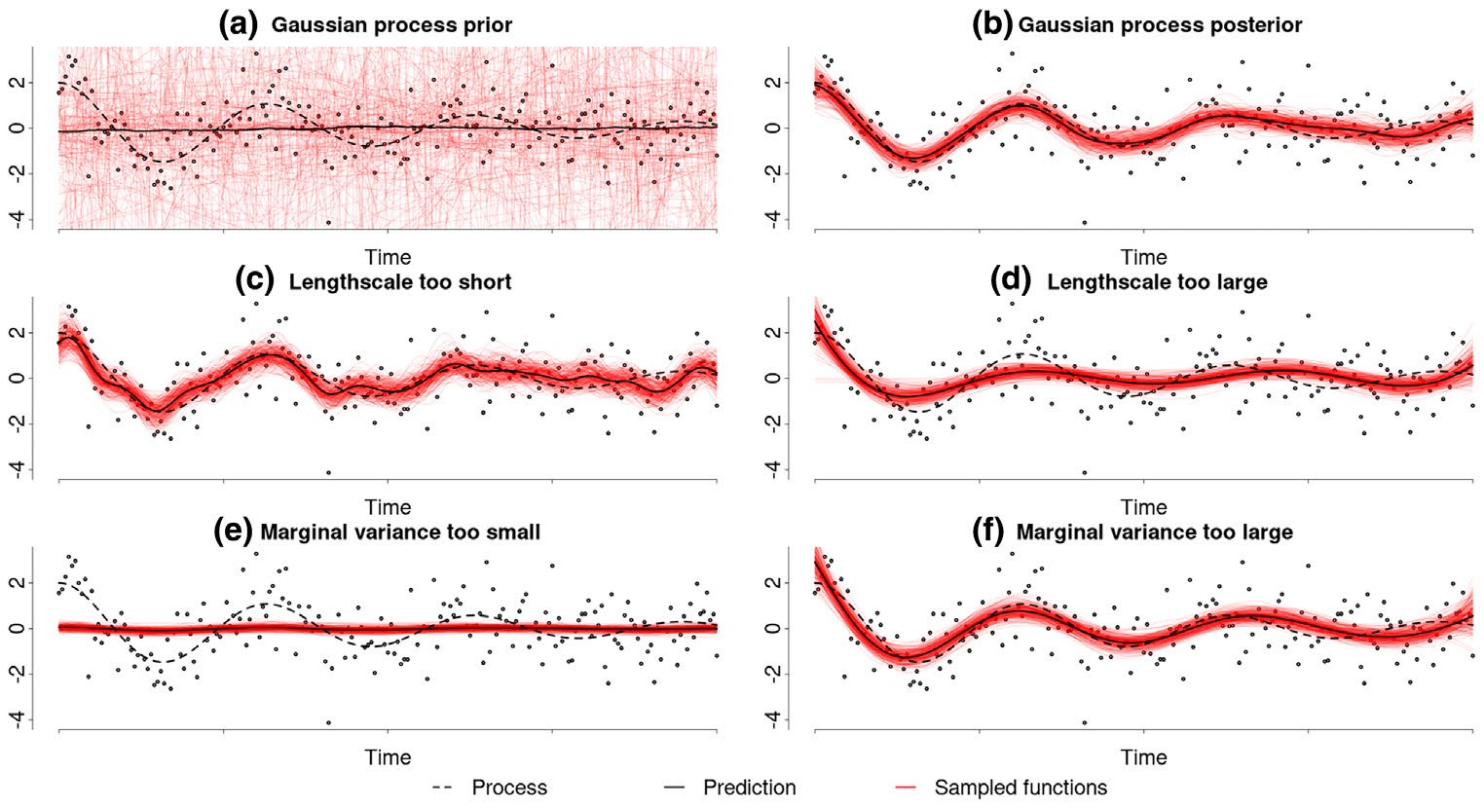
Demonstration of a Gaussian process regression. This figure shows how GP regression (solid black) estimates the underlying process (dotted black). Panel (a) shows a sample from a GP prior with a squared‐exponential kernel and half‐normal hyperparameter priors. Panel (b) shows a sample from the corresponding GP posterior distribution. Panels (c, d) illustrate the effect of fixing the lengthscale of the GP prior to values that are too small or too large, respectively. Panels (e, f) illustrate the impact of fixing the marginal variance of a GP prior to values that are too small or too large, respectively.

This posterior distribution represents an updated belief about which functions describe the latent process well, making it possible to draw inferences about the process (Rasmussen & Williams, 2006). Figure 3a shows a sample of functions (in red) drawn from a GP prior. This prior was combined with a Gaussian likelihood to obtain a sample from the corresponding posterior distribution of the latent process, shown in Figure 3b. This sample of functions from the posterior GP can be used to obtain pointwise estimates and credible intervals for the underlying process.

A GP distribution is defined by a mean function m(t) and a covariance function cov(t, t). These functions are continuous extensions of the mean vector and covariance matrix of a multivariate normal distribution. Together, they define the mean and covariance of the values that the functions described by the GP assume at any finite set of time points. In most applications, the mean function is set to zero when no specific prior knowledge is available. This results in a GP prior without an average trend, as illustrated in Figure 3a. However, this does not restrict the posterior mean to zero, as shown in Figure 3b, where the posterior mean closely follows the underlying process. Instead, it indicates a lack of prior information about its deviations from zero. However, if there is prior information available about the trend of the non‐linear process (such as in a developmental context, where a learning curve could be expected to have a general upwards trend) it would be possible to include this in the GP by using, for example, a linear or quadratic mean function.

**FIGURE 4:**
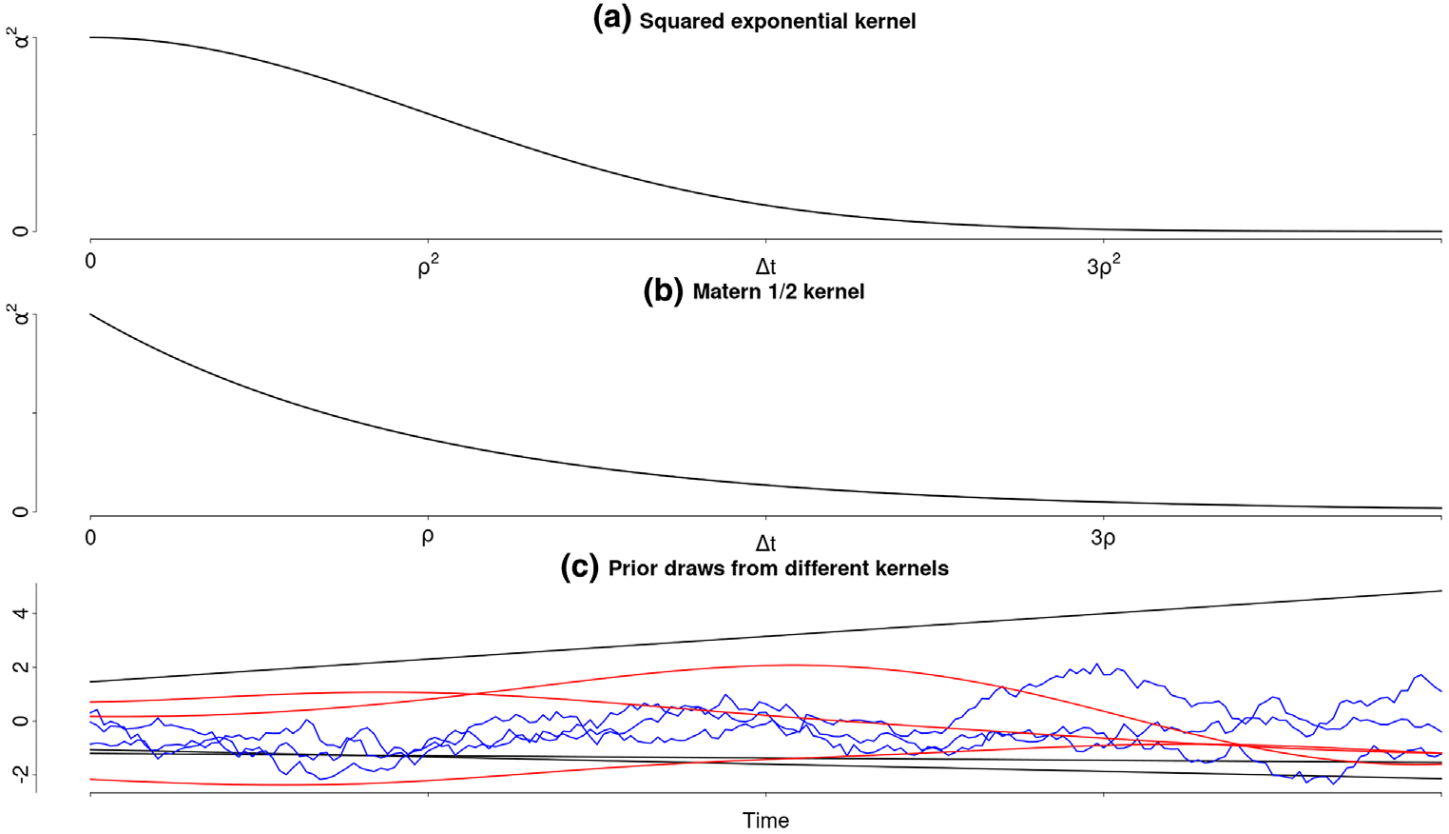
Illustration of different Gaussian process kernels. Panel (a) depicts the squared‐exponential kernel. Panel (b) depicts the Matérn 1/2 kernel. Lastly, panel (c) shows three functions drawn from GP priors with different covariance kernels. The black lines show functions drawn from a GP with a dot‐product kernel, the red lines show functions drawn from a GP with a squared exponential kernel, and the blue lines show functions drawn from a GP with a Matérn 1/2 kernel.

The covariance function is typically based on a kernel function, similar to the kernel used in the LPR. The types of processes that can be captured by a GP regression primarily depend on the chosen form of the covariance kernel. This is because the covariance kernel largely dictates the behaviour of the functions described by the GP prior. Consider, for example the dot‐product kernel:

(8) $$\mathrm{cov}(t_{i},t_{j})=k_{\mathrm{GP}}(t_{i},t_{j})=\sigma_{\alpha }^{2}+\sigma_{\beta }^{2}t_{i}t_{j}$$

which generates a Gaussian process that describes linear functions and is equivalent to a Bayesian linear regression (Figure 4c, black). In this case, the variances $\sigma_{\alpha }^{2}$ and $\sigma_{\beta }^{2}$ are the prior variances of the intercepts and slopes of the linear functions defined by the GP.

The default covariance kernel in most standard software is the squared exponential (Figure 4a), which produces a non‐linear Gaussian process that is covariance stationary (such that the described covariances between the function values depend only on their distance) and very smooth (Figure 4c, red). This kernel was used for the GPs in Figure 3a,b. Using the squared exponential covariance kernel imposes a stricter smoothness assumption on the underlying process than the smoothness assumption introduced for LPR. However, many other covariance kernels are available, each resulting in GPs with different behaviours and assumptions about the underlying process. A notable example is the Matérn class of kernels, which relaxes the strict smoothness assumption made by the squared exponential kernel. This makes it possible to model rougher processes, as illustrated by the blue samples in Figure 3c. The Matérn 1/2 kernel (Figure 4b) may be particularly relevant for psychological applications, as it describes the covariance structure of a continuous‐time autoregressive process. We will, however, focus on the squared exponential kernel, which is the default in most standard software.

As was the case with the LPR, the kernels (and thus the covariance functions) used in a GP have parameters called hyperparameters, which determine the scale and variability of the estimated non‐linear process. Most common covariance kernels (such as the introduced squared exponential or the Matérn class kernels) build on a characteristic lengthscale ρ and a marginal standard deviation parameter α. The characteristic lengthscale effectively determines the wiggliness of the estimated process by quantifying how quickly the covariance decreases with increasing distances between time points. Such that a smaller lengthscale corresponds to a wigglier process. Since GPs typically use a single lengthscale for the entire process, they assume a constant level of wiggliness throughout the underlying process. The marginal standard deviation describes the spread of the functions described by the GP at any point in time.

#### 2.2.2 Practical implementation

When fitting a GP regression, choices need to be made regarding the mean function and the covariance kernel, as well as the lengthscale and marginal variance of the kernel. In the absence of prior knowledge about systematic deviations from zero, the mean function is typically set to zero. However, other mean functions can be chosen to incorporate specific domain knowledge. The squared exponential kernel is the default choice in most standard software because it produces smooth estimates. However, kernels from the Matérn class can be used instead, if less smooth estimates are desired (such as the blue functions depicted in Figure 4c). The choice of a specific kernel can be informed by prior theory, specific smoothness requirements or in a data‐driven manner by model comparison methods such as Bayes factors or cross‐validation.

The lengthscale determines how quickly the covariance between function values decreases over time. Therefore, it controls the wiggliness of the estimated process. It can be estimated by optimizing a cross‐validation criterion or the models marginal likelihood, in order to prevent over‐ or underfitting the data. A lengthscale that is too small leads to an estimated process that is too wiggly and overfitting (Figure 3c), whereas a lengthscale that is too large results in underfitting (Figure 3d). While the lengthscale of a GP can be optimized in a similar way to the hyperparameters of the other methods presented in this paper, we recommend using a full Bayesian approach that assigns a prior distribution to the lengthscale. An advantage of this full Bayesian approach is that it provides uncertainty estimates, not only for the process itself, but also for the lengthscale through its posterior distributions. In cases where no prior information is available about the wiggliness of the process, this prior can be vague to assume that all parameter values are equally likely a priori. Moreover, in practice, it is often helpful to standardize the predictor and outcome variables when setting priors. This removes the original scaling of variables, such as IQ scores and allows priors to be defined independently of the original variable mean and variance.

The marginal variance of a GP determines the spread of the functions it describes at any point in time. It can be estimated in the same ways as the lengthscale. Figure 3e shows that a marginal variance that is too small leads to a GP prior that is too narrow and unable to adequately capture the process, which causes underfitting. However, a marginal variance that is too large does not result in overfitting to the same degree, as shown in Figure 3f.

The brms package (burkner‐brms 2017) provides an accessible implementation of Gaussian process regression using the model syntax

brm(y ∼ gp(time))

This code automatically uses a squared‐exponential kernel and assigns vague priors to the hyperparameters. Specific informative hyperparameter priors can be added to the brm function using the optional prior argument. The GP prior used in Figure 3a assigns half‐normal priors with standard deviations of one to the lengthscale and five to the marginal variance and standardizes the predictor variable. The brms model syntax also makes it straightforward to combine multiple GPs in a model or to incorporate parametric components, such as a linear slope. After fitting a GP using the code presented above, researchers obtain posterior samples for the marginal variance and the lengthscale parameter. The posterior distribution for the lengthscale, in particular, can be understood as an estimate of the wiggliness of the process. Additionally, the posterior samples for the hyperparameters can be used to obtain posterior samples for the underlying process itself, which are depicted Figure 3a. These posterior samples can be used to construct point estimates and credible intervals for the process and any of the hyperparameters.

### 2.3 Generalized additive models

#### 2.3.1 Statistical theory

Generalized additive models (GAMs) are a class of semi‐parametric models that can be seen as an extension of regression models that does not use variables as predictors of an outcome but so‐called smooth terms:

(9) $$Y_{t}=\beta_{0}+\overset{I}{\underset{i=1}{\sum }}\beta_{i}(t_{i}),$$

where each smooth term $\beta_{i}$ reflects a non‐linear function of a predictor (e.g. time). A GAM combines these different smooth terms into an overall estimate of a non‐linear process (Hastie & Tibshirani, 1999; Wood, 2006, 2020). The smooth terms $\beta_{i}$ are inferred from the data through a method called smoothing splines. These smoothing splines take the form of a basis function regression, which does not use the raw predictor values directly. Instead, it combines multiple predefined functions (called basis functions) of the predictor $R_{ki}(t_{i})$, such as polynomial basis functions of different degrees, along with their weights $\alpha_{ki}$ in a regression model:

(10) $$\beta_{i}(t_{i})=\overset{K}{\underset{k=1}{\sum }}\alpha_{ki}R_{ki}(t_{i})$$

Generally, using more basis functions (and thus coefficients) results in a smoothing spline (and thus smooth term) that is more flexible, which means that it can fit the data closer during the estimation. Smoothing splines rely on using enough basis functions to be able to overfit the data, which ensures that they are flexible enough to capture the underlying process accurately.

**FIGURE 5:**
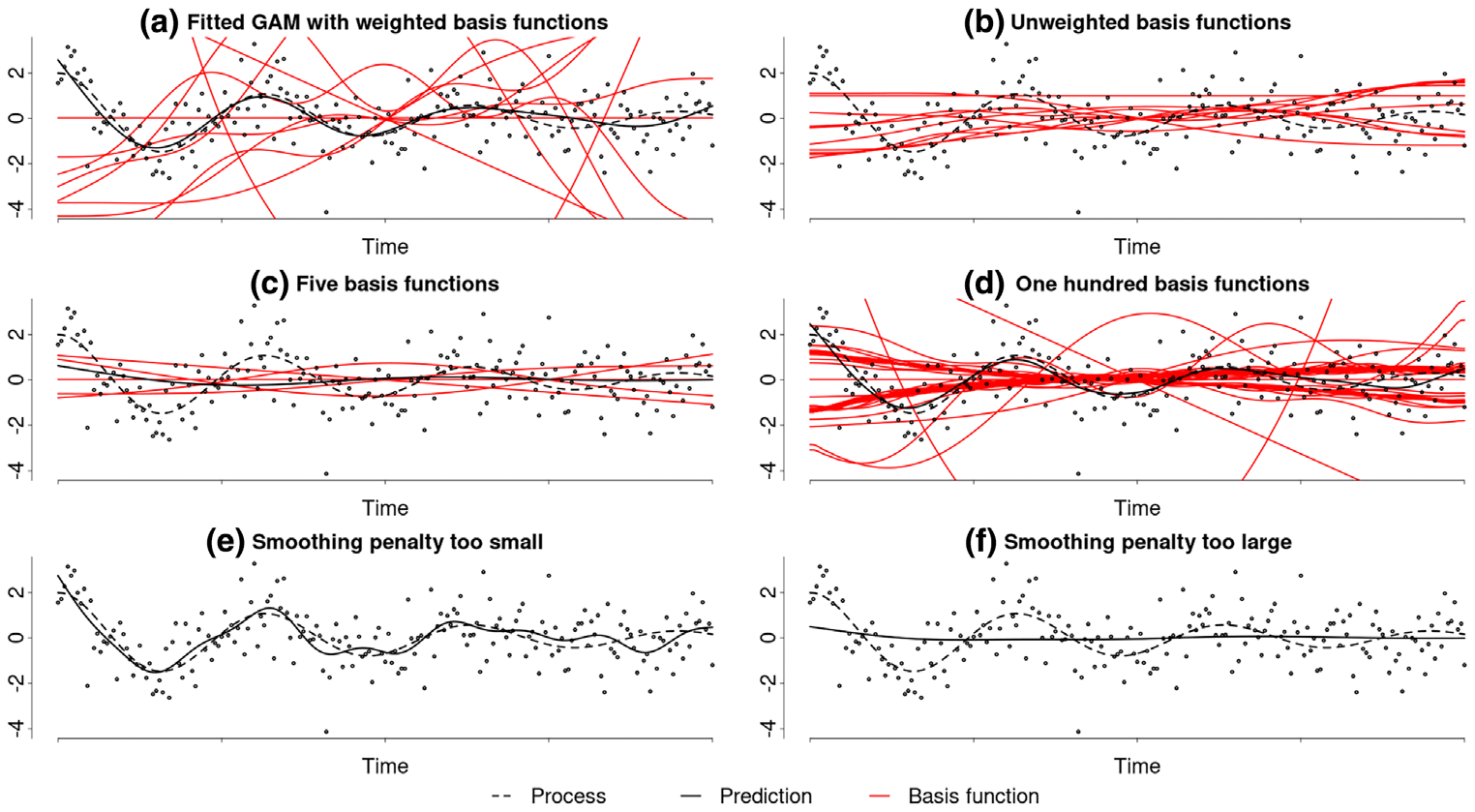
Demonstration of the construction of a GAM. Panel (a) shows a fitted GAM with a single smooth term for time. The 10 weighted thin‐plate spline basis functions (red) are summed to produce an estimated of the underlying. The unweighted basis functions are depicted in panel (b). Panels (c, d) illustrate the effect of fitting a GAM with too few or too many basis functions, respectively. Similarly, panels (e, f) illustrate GAMs with a smoothing penalty that is too small or too large, leading to over‐ or underfitting, respectively.

In summary, GAMs use a sum of smooth terms to model the outcome variable, where each smooth term is estimated using a smoothing spline. These smoothing splines build on basis functions such that a greater amount of basis functions yields a more flexible smoothing spline. Figure 5a shows a GAM composed of a single smooth term that was fit to the example process. The 10 weighted basis functions that constitute the smoothing spline are shown in red. While GAMs can combine multiple smooth terms, in this paper, we will focus on the simplest GAMs consisting of a single smooth term.

Smoothing splines can be constructed using different families of basis functions, called spline bases. The most common basis is thin‐plate splines, which are a generalization of cubic regression splines (Wood, 2006). Thin‐plate splines are composed of basis functions, which are automatically generated and increasingly wiggly. Each basis function is defined over the entire range of the data (Figure 5b) and if there are as many basis functions as data points, thin‐plate splines can perfectly interpolate the data. When estimating the coefficients (i.e. weights) of the basis function regression, smoothing splines use an additional penalty term, similar to those used in a lasso or ridge regression, to control how closely the smooth term fits the data during the estimation. Optimizing the weight of this smoothing penalty balances the complexity and fit of the smooth term, ensuring the model captures the underlying process accurately without overfitting (Gu, 2013; Wahba, 1980).

Unlike the LPR or GP, GAMs lack a specific parameter such as the bandwidth or lengthscale that directly reflects the wiggliness of a process. Instead, GAMs indicate the wiggliness of each smooth term through their respective effective degrees of freedom (EDF), which measure the complexity of each smooth term. Similar to the degrees of freedom in a regression model, the EDF are tied to the number of coefficients and, therefore, the number of basis functions in a smooth term. However, due to the penalty applied in GAMs, many of these coefficients are constrained and cannot vary independently. This reduces the effective degrees of freedom for each smooth term. Depending on the weight of the penalty, the EDF can range from one (indicating a linear smooth) to the total number of basis functions, with larger EDF values generally reflecting a wigglier smooth term. However, unlike LPR and GPs, GAMs have the advantage that they do not assume constant wiggliness in the underlying process. This flexibility arises because the penalty term in a GAM only captures the overall wiggliness of the estimated process^4^ and, thus, does not constrain the wiggliness to be constant over time. For example, an estimated process may have low wiggliness at the beginning and high wiggliness at the end, yet still have the same overall penalty as another estimate with consistent moderate wiggliness throughout the entire range.

The standard error estimates provided by GAMs commonly assume that the weight of the penalty term is fixed, whereas in reality it is most frequently optimized in a data driven manner. Because of this, the standard errors tend to underestimate the uncertainty associated with the process estimate. However, simulations have shown that, except when estimating process that are close to linear, the standard errors remain reasonably accurate, resulting in confidence intervals with close to nominal coverage proportions (Marra & Wood, 2012). Additionally, the accuracy of the standard errors could be improved through, for example, Bayesian estimation, which accounts for the uncertainty in the penalty weight (Wood, 2006).

#### 2.3.2 Practical implementation

When applying GAMs, researchers need to make three decisions regarding the spline basis, the number of basis functions and the weight of the smoothing penalty. For most applications, thin‐plate splines are an optimal default choice (Wood, 2006). The number of basis functions should be as large as possible (corresponding to the number of data points) to ensure that the smoothing spline is sufficiently flexible to capture the underlying process accurately. Figure 5c shows a smoothing spline with too few basis functions, leading to underfitting. However, using too many basis functions does not similarly result in overfitting (Figure 5d), as the smoothing penalty can control the excessive model complexity. Nevertheless, a large number of basis functions can make models computationally expensive or infeasible for larger data sets. To address this, dimension reduction techniques similar to principal component analysis can approximate a smoothing spline solution using fewer basis functions (Wood, 2003). In this approach, the number of basis functions should be sufficient to capture the underlying process while remaining computationally practical. This procedure is automated in standard software (Wood, 2011); however, it is not used in this paper.

Lastly, the smoothing penalty weight must be optimized to avoid over‐ or underfitting. A penalty that is too small results in a smoothing spline that is overly flexible and prone to overfitting (Figure 5e). Conversely, a penalty that is too large yields a spline that is not flexible enough, leading to underfitting (Figure 5f). The smoothing penalty weight is typically optimized through data‐driven methods, such as directly maximizing the likelihood or using cross‐validation.

GAMs are implemented in the mgcv package (Wood, 2011). The model shown in Figure 5b was implemented using the code

gam(y ∼ s(time, bs = 'tp', k = 10))

which fits the observed data with a single smooth term for time. The syntax specifies that the smooth term should be composed of 10 thin‐plate spline basis functions, although the number of basis functions can also be set to a larger value or determined automatically. The smoothing penalty weight is by default optimized by minimizing the generalized cross‐validation error. Similar to GPs, the mgcv model syntax allows for the creation of more sophisticated models by combining multiple smooth terms or incorporating parametric components.

Fitting a GAM with the code provided above returns an estimate of the underlying process along with corresponding credible intervals, which are depicted in Figure 5a. Additionally, researchers obtain the EDF for each smooth term separately. While the EDF are useful for assessing the wiggliness of a smooth term, it is important to note that they are not model parameters. Consequently, unlike GPs, GAMs do not provide a measure of uncertainty for the wiggliness of the process. The mgcv implementation of GAMs also includes significance tests to determine whether a given smooth term is effectively zero. Hence, a significant test result may indicate any linear or non‐linear process that is not a constant line at zero (Bringmann et al., 2017; Wood, 2013). Lastly, it is possible to extract the individual basis function coefficients from a fitted GAM.

### 2.4 Key differences between the methods

A key difference between the three non‐linear methods introduced in this paper is the information they provide about the wiggliness of the underlying process. Each method measures different aspects of the wiggliness and the interpretation of the wiggliness estimates of each method depends on the chosen respective configurations. For instance, the interpretation of the bandwidth of an LPR changes depending on the selected polynomial degree and kernel. Similarly, the interpretation of the lengthscale of a GP regression changes depending on the chosen mean function and covariance kernel. This makes it almost impossible to compare the wiggliness estimates between the presented methods and even between different configurations of the same method. Other important differences between the methods are summarized in Table 1.

**TABLE 1:** A comparison of LPR, GP regression and GAMs.

	LPR	GP	GAM
Advantages	Intuitive theory Completely data driven	Interpretable parameters Natural uncertainty quantification Can incorporate (incomplete) prior theory Follows standard Bayesian analysis steps	Intuitive theory Some interpretable parameters Can incorporate (incomplete) prior theory
Disadvantages	Least interpretable parameters Biased for most processes No uncertainty estimate for the wiggliness	Difficult to specify in practice	No uncertainty estimate for the wiggliness Standard errors may be underestimated
Required choices	Polynomial degree Kernel Optimization criterion	Covariance kernel Mean function Hyperpriors	Spline basis Number of basis functions Optimization criterion
Key assumptions	P‐times differentiable process Constant wiggliness	Assumptions depend on chosen specifications	Smooth process Homoscedasticity
Estimation	OLS	Bayesian, Marginal likelihood maximization	OLS, MLE, Bayesian
Key sources of information	Fan and Gijbels (2018)	Rasmussen and Williams (2006)	Wood (2006)

## 3 SIMULATION

### 3.1 Problem

A simulation study was conducted to assess the effectiveness of the introduced methods in recovering different non‐linear processes that may be encountered in EMA research (Figure 1). In this simulation, the three methods were not only compared against each other but also to a polynomial regression model (the current most used method to model non‐linear trends in psychology) and to parametric models that accurately specify the respective non‐linear processes. These (data‐generating) parametric models were added to serve as a benchmark for the non‐linear process recovery. To apply the introduced methods in line with how they were introduced and within the constraints of available software implementations, the simulation focused on a univariate single‐subject design. Hence, the simulated data represented repeated measurements of a single variable for one individual.

### 3.2 Design

To conduct the simulation with processes that might be encountered in real EMA studies, we selected the exemplar processes illustrated in Figure 1 as a basis. These include two growth curves (modelled as an exponential and a logistic growth curve), a mean‐level switching process (modelled as a cusp catastrophe) and a self‐regulatory process (represented by a damped oscillator).

**FIGURE 6:**
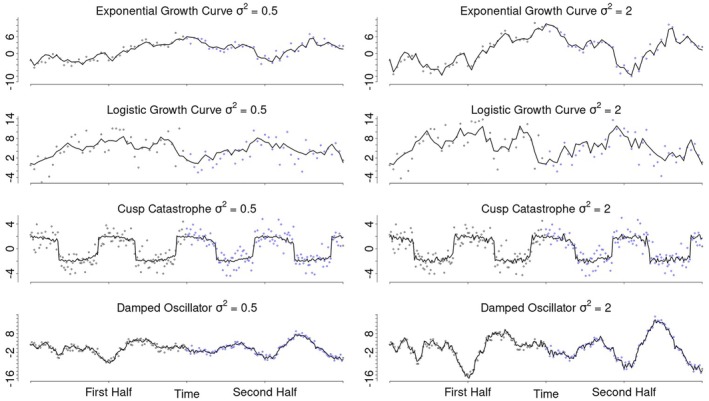
Simulation conditions. This figure illustrates the different conditions that were manipulated in the simulation. It shows a possible realization of each exemplar process with a dynamic error variance of 0.5 (left) and 2 (right). Further, it shows how the sampling period was manipulated by sampling either only over the first half (black dots) or over the entire period of each process (black and blue dots). Lastly, the sampling frequency was manipulated. The top four panels display samples with one observation per time step, whereas the bottom four panels display samples with three observations per time step.

**FIGURE 7:**
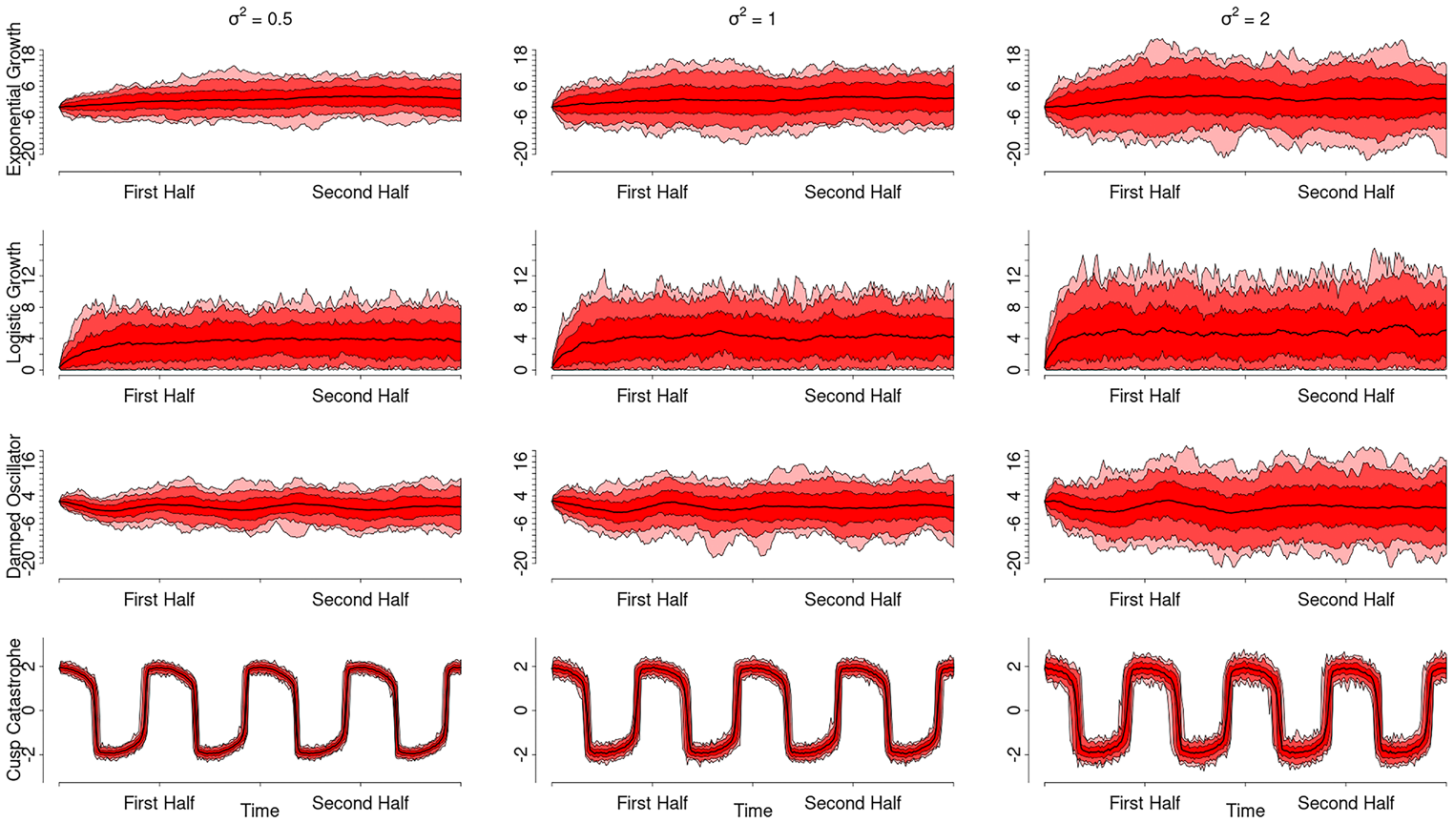
Pointwise means and standard deviations of the simulated stochastic processes. This figure depicts the pointwise means and standard deviations of 100 data sets simulated from each of the stochastic processes, with dynamic error variances of 0.5, 1 and 2. The solid black line depicts the mean of all simulated data sets at each time point. The shaded red areas depict 67%, 95% and 99.7% of the simulated data around the mean at each time point.

Since real live processes are likely affected by external influences (i.e. context effects), we account for these influences in the simulation by adding dynamic errors to each process. These dynamic errors reflect external perturbations (or other forms of unaccounted influence) to the latent construct that are carried forward in time. For instance, if a participant experiences an unusually pleasant conversation that elevates their true positive affect, this change represents an error effect if it is not accounted for by the model. However, since the true positive affect level has increased, future measurements will be influenced due to, for example, emotional inertia. As such, these dynamical errors make the processes rougher/less smooth (i.e. non‐differentiable; Figure 6). The degree of roughness was controlled by the variance of the dynamic errors. To demonstrate the varying roughness of the processes, Figure 6 presents a possible realization of each process with a dynamic error variance of 0.5 (left) and with a variance of 2 (right). We considered variances of 0.5, 1 and 2 reasonable relative to the process range. Additionally, Figure 7 displays the means and variances of 100 data sets sampled from each of the stochastic processes for varying dynamic error variances. Importantly, we intentionally omitted a condition without dynamic errors from this simulation, as dynamic errors are reasonably expected to be present in all psychological ILD.

Additionally, we varied both the sampling period (i.e. the duration over which the process is measured) and the sampling frequency (i.e. the number of measurements taken during that period) for each process. Since the data in the simulation is generated, it does not have an inherent time scale. This means that one time step in the simulated data could represent an hour, a day or a year and that the time scaling of the simulated processes is only meaningful relative to the scale at which each process exhibits its characteristic behaviour. For instance, if the chosen time scale is too long, both growth curves (Figure 1a,b; with the chosen parameters) would display a brief period of growth followed by a long, nearly flat phase approaching the asymptote. To ensure consistency, all processes were simulated to display their characteristic behaviour over the same period.

To simulate measuring each process for a shorter or longer time, thus capturing different behaviours of each process, data was either generated over only the first half (Figure 6, black dots) or over the entire period (Figure 6, black and blue dots). For example, sampling the two growth curves only over the first half of the period would mean that the process is not measured close to the asymptote.

Lastly, we manipulated the sampling frequency by varying the number of equidistant measurements taken per time step. As a result of this simulation design, the overall sample size in each condition is determined by the combination of the sampling period and the sampling frequency. For example, increasing the sampling frequency within a fixed period by a factor of two halves the interval between consecutive measurement points and, in turn, approximately doubles the overall sample size. Similarly, doubling the sampling period also doubles the overall sample size without changing the distance between measurements. Sampling frequencies of one, two and three observations per time step produced sample sizes typical of EMA studies, ranging between 43 and 253 total observations (Wrzus & Neubauer, 2023). This is illustrated in Figure 6 where the top four panels show examples with one observation per time step, whereas the bottom four panels illustrate three observations per time step, simulating a scenario where measurements are taken three times more frequently. Overall, this resulted in sample sizes ranging between 43 observations (one observation per time step for half the period) to 253 observations (three observations per time step for the entire period).

### 3.3 Hypotheses

Since the parametric models matched the true data‐generating models, we expected them to infer the underlying processes most accurately, serving as a benchmark for comparison with the other methods. We also anticipated that the (global) polynomial regression would infer all processes less accurately than the three introduced approaches because of the limitations outlined in the introduction. However, we did not have specific hypotheses regarding the relative performance of the three non‐ and semi‐parametric methods.

Additionally, we anticipated that all methods, except the parametric models, would struggle to accurately infer the cusp‐catastrophe process, as each method produces continuous estimates that may be inadequate for capturing the apparent jumps in the process. Similarly, because all methods (except the parametric models) produce smooth estimates using the default configurations in which they are most often applied and implemented in standard software, we expected the performance of all methods to decline with larger dynamic error variances.

Our expectations regarding the sampling period were more nuanced. The LPR and GP assume that the wiggliness (as defined by each non‐parametric method, respectively) of the process remains constant over time. However, all four processes exhibit changes in wiggliness. For example, the wiggliness of the exponential and logistic growth functions and the damped oscillator decreases monotonically, while the cusp‐catastrophe's wiggliness fluctuates cyclically (i.e. low wiggliness during plateau phases and high wiggliness during jumps). Therefore, we hypothesized that longer sampling periods for the exponential and logistic growth curves and the damped oscillator would reduce the inference accuracy of the LPR and GP as the single bandwidth or lengthscale parameter becomes increasingly inadequate to capture the changing wiggliness over time. Furthermore, the LPR was not expected to benefit from the additional data provided by measuring the process for a longer time, since it relies mostly on data in its local neighbourhood during the estimation. Therefore, extending the sampling period beyond these local neighbourhoods was not expected to increase the inference accuracy. In contrast, the GAMs and parametric models, which do not assume constant wiggliness and utilize the entire data set during the estimation, were expected to infer all processes more accurately with larger sampling periods.

Lastly, we predicted that increasing the sampling frequency would generally improve the inference accuracy across all methods by providing more information about the underlying processes.

### 3.4 Outcome measures

To evaluate and compare the performance of the different analysis methods, we focused on three outcome measures: the mean squared error (MSE), the generalized cross‐validation criterion (GCV) and the confidence interval coverage. To assess how effectively each method captured the non‐linear process at the observed time points, we calculated the MSE between the estimated and generated process values. Additionally, to evaluate how well each method would predict omitted process values within the process range, we computed the GCV (Golub et al., 1979) criterion for each method and data set. The GCV is a more computationally efficient and rotation‐invariant version of the ordinary leave‐one‐out cross‐validation criterion with the same interpretation. Both, the GCV and the leave‐one‐out cross‐validation criterion, describe how accurately the model predicts omitted data points within the design range, which provides information about how well the model interpolates the process. However, in contrast to the ordinary leave‐one‐out cross‐validation, the GCV does not require each model to be refit to many data subsets. Instead it utilizes that for all presented methods, a linear function exists that maps the observed data points to the predicted process values (i.e. the influence, projection or hat matrix). This linear function can be used to analytically calculate how the predicted values would change if any of the data points were removed from the model, which makes it possible to calculate a cross‐validation criterion, without the computationally intensive refitting of the model.^5^

To evaluate the uncertainty estimates provided by each method, we recorded whether the true generated process was located within the confidence or credible intervals at each time point. Subsequently, the average confidence interval coverage proportion for each method and data set was obtained by averaging over all time points. Given that all confidence or credible intervals were set at a 95% confidence level, the expected coverage proportion should ideally be close to 95%. Due to Monte Carlo error in the simulation ($\max(se_{\mathrm{CIC}})\approx 0.03$), individual average confidence interval coverages are expected to deviate from the ideal 95%. Because of this, average coverage proportions between 89% and 100% were also deemed acceptable. Average coverage proportions below 89% may indicate (but may not only be due to) underestimated uncertainty.

### 3.5 Data generation

Each process in Figure 6 was represented as a generative stochastic differential equation model. These dynamic models describe the relationship between the process's current value and its instantaneous rate of change. Combined with information about the initial state of the process this makes it possible to describe the entire process indirectly. For instance, the stochastic differential equation model used to represent the introduced logistic growth process can be expressed as follows:

(11) $$dy=ry(1-\frac{y}{k})dt+\sigma dW_{t}$$

The first half of this model defines the deterministic dynamics of the process. It relates the rate of change of y to its current value and to how far away the current value is from the asymptote k through a growth rate constant r. The second part of the model accounts for the dynamic errors in the form of a Wiener process. The Wiener process is a continuous non‐differentiable stochastic process, which describes normally distributed dynamic errors over any given discrete time interval. These errors have a mean of zero and a variance depending on the length of the time interval and σ, making them optimal for this simulation. Importantly, these dynamic errors continuously influence the rate of change of the process and are propagated forward in time through the deterministic dynamics of the model. Simulating the other processes was achieved by simply using other equations for the deterministic dynamics in Equation (11). The precise equations used for the other methods are detailed in the Appendix S1.

The resulting processes were then simulated using the Euler‐Maruyama method, which approximates stochastic differential equation systems with an arbitrarily high accuracy by linearizing them over small discrete time intervals. The resulting high‐resolution data were then subsampled to achieve the desired sampling frequency. Finally, measurement errors were added to the latent process data at each time point independently from a standard normal distribution, generating the final sets of observations.

Based on an initial pilot sample of 30 data sets per condition, we determined the number of replications needed to achieve a Monte Carlo standard error of less than 0.03 for the confidence interval coverage for all methods, models and simulation conditions (Siepe et al., 2023). These Monte Carlo standard errors reflect the expected variation in the outcome statistics due to random processes within the simulation. This analysis showed that 100 replications per condition would result in maximal Monte Carlo standard errors of approximately $se_{\mathrm{MSE}}\approx 0.05$, $se_{\mathrm{GCV}}\approx 0.38$, and $se_{\mathrm{CIC}}\approx 0.03$.

### 3.6 Model estimation

After simulating the data, all introduced methods were applied to each data set using the statistical software R (R Core Team, 2024). The specific configurations used for each method were chosen to reflect how each method is most commonly applied in practice and not to optimally infer the simulated, and thus known, processes. First, the LPR was estimated using the nprobust package (Calonico et al., 2019), which can correct for the bias inherent in LPRs. Second, GPs were estimated in STAN^6^ (Gabry et al., 2024) with a zero mean and a squared exponential covariance function, following common practice. Third, GAMs with a single smooth term for time were fitted using the mgcv package (Wood, 2011). The polynomial regressions were estimated using base R, with correlated (i.e. standard) polynomial terms and data set specific polynomial degrees. Finally, the parametric stochastic differential equation models corresponding to the true data‐generating models were estimated using the Dynr package (Ou et al., 2019). After fitting each model to the data, they were used to obtain point and interval estimates (i.e. 95% confidence and credible intervals) for the latent process at each time point. A detailed description of each model fitting procedure is provided in the Appendix S1. Further, if any models failed to converge during the simulation, the corresponding outcome measures were excluded from the following analyses.

The R code used to simulate the data, fit the models, obtain the results and generate the figures is available in Appendix S1. Additionally, a web application is provided in https://jan‐ian‐failenschmid.shinyapps.io/modeling_non_linearity_app/, allowing readers to further inspect and explore the results presented in the paper. The web application also enables users to simulate data based on any of the processes discussed in the paper and fit some of the computationally less intensive models.

### 3.7 Results

In the simulation, a small proportion of GP regressions (1.76%) and parametric models (28.60%) did not converge.^7^ This is most likely due to the small sample sizes considered and the automated model fitting in the simulation. Most notably, the parametric model was not able to infer the cusp catastrophe from the small simulated data sets due to the complexity of the model. The performance measures of the methods that did not converge were removed from the following analysis. Additionally, the parametric models appear to have overfit for a small number of data sets from all processes. The complete simulation results and data are available in the Appendix S1.

#### 3.7.1 Capturing the non‐linear process

**FIGURE 8:**
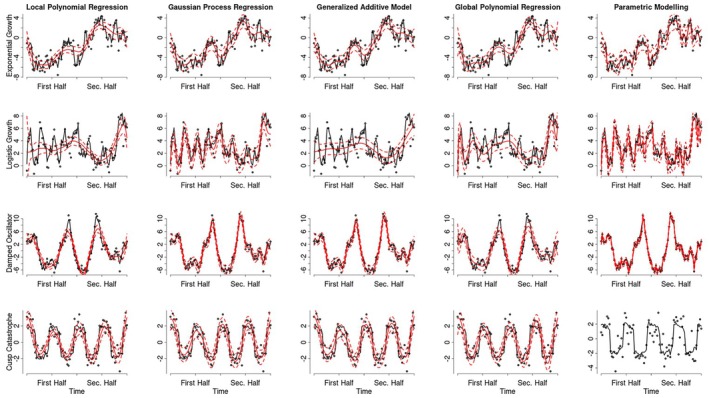
Example processes inferred by each of the introduced methods. This figure shows how each of the introduced methods inferred an example of each of the processes from the simulation. In this figure, the black line shows the true simulated process, while the solid red line shows the estimated process. The dashed red lines around the estimated process display 95% confidence or credible bands. No estimates are displayed in the last panel since the parametric models did not converge for any of the simulated data sets of the cusp catastrophe model.

It is noteworthy that the point estimates produced by all considered methods were able to visually follow the simulated processes adequately. Figure 8 illustrates an example of each process being inferred by all methods. While the predicted means produced by each method all follow the overall trajectory of the processes, several observations can be made based on this figure. First, the local and the global polynomial regression appear to underfit (i.e. oversmooth) for some of the processes. Second, while the global polynomial regression generally underfit the processes, it appears to overfit near the boundary (i.e. the beginning and end of the time series) resulting in excessive uncertainty. This is a known problematic behaviour of the global polynomial regression. Lastly, all methods appear to struggle with accurately capturing the cusp‐catastrophe, especially its nearly stable periods, often producing estimates that resemble sine functions. However, there is considerable variation and overlap in the accuracy and behaviour of the different methods across the various data sets, which highlights the need for a more formal analysis of the performance of each method.

To summarize the high dimensional results efficiently, two separate ANOVAs were fitted to the MSE and GCV values, including all possible main and interaction effects. Table 2 presents all effects for which the partial‐$\eta^{2}$, indicates at least a small effect size for either the MSE or the GCV. The most influential effects for both the MSE and GCV belonged to the analysis method, the data‐generating process and the dynamic error variance. The main effects of the sampling period and sampling frequency were considerably smaller and comparable in magnitude to some of the first‐degree interaction effects. The following sections will focus on describing the most important of these effects. A comprehensive overview of all effects in the models can be found in the Appendix S1.

**TABLE 2:** Effect sizes from the MSE and GCV ANOVAs.

Effect	Partial‐$\eta^{2}$ MSE	Partial‐$\eta^{2}$ GCV
Method	.41	.25
Process	.55	.40
SP	.17	.08
SF	.15	.23
DEV	.56	.48
Method:Process	.21	.12
Method:SP	.15	.08
Process:SP	.06	.04
Method:SF	.05	.01
Process:SF	.01	.04
Method:DEV	.17	.10
Process:DEV	.27	.23
SP:DEV	.02	.02
SF:DEV	.01	.05
Method:Process:SP	.07	.05
Method:Process:SF	.03	.01
Method:SP:SF	.01	.00
Method:Process:DEV	.07	.05
Method:SP:DEV	.02	.02
Process:SP:DEV	.01	.01
Process:SF:DEV	.00	.02
Method:Process:SP:DEV	.01	.02

*Note*: This table shows all effects from the MSE and GCV ANOVA that had at least a small effect partial‐*η* ^2^> = .01 on either outcome.

Abbreviations: DEV, dynamic error variance; SP, sampling period; SF, sampling frequency.

**FIGURE 9:**
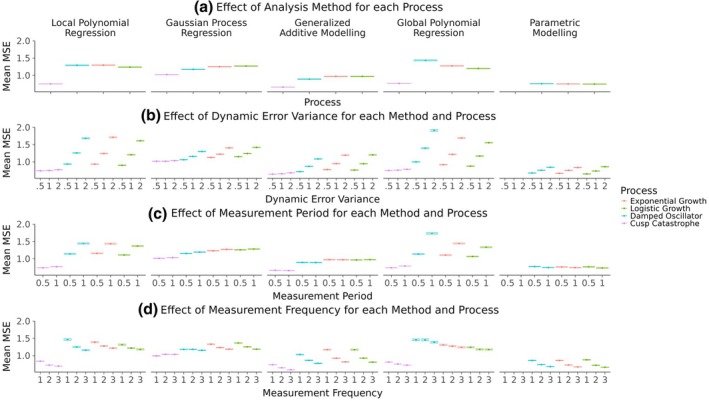
Mean MSE effects across all processes, analysis methods and simulation conditions. Panel (a) shows the effect of the analysis method for each process. The other three panels show the effects of measurement period (b), measurement frequency (c) and dynamic error variance (d) for each analysis method and latent process.

The mean MSE results are depicted in Figure 9. Panel (a) shows the average MSE with which each method inferred each of the processes, when averaging over all other conditions. There appear to be clear differences in the average MSE with which each method infers all processes on average, illustrating the main effect of the analysis method. Specifically, the parametric modelling showed the lowest average MSE, followed closely by the GAMs, whereas the GP regression, LPR and the polynomial regression had larger average MSE values. Similarly, the main effect of the process can be seen in that some processes were inferred with a lower average MSE by all methods. Most notably, the cusp catastrophe was inferred with lower MSE values than the other processes across all methods. Lastly, panel (a) also depicts the interaction effect between the methods and the processes, since the mean MSE differences between the different analysis methods are not equal across the different processes. For example, the Gaussian process regression displayed a smaller mean MSE than the polynomial regression for the damped oscillator process, but a larger mean MSE for the cusp catastrophe.

Figure 9b illustrates the effect of increasing dynamic error variances for all methods and processes (averaged over all sampling periods and frequencies), which is the largest main effect among the simulation conditions. A larger dynamic error generally led to larger average MSE values. However, this effect was considerably less pronounced for the parametric models, GPs, GAMs and the cusp‐catastrophe process, highlighting the interaction between the dynamic error variance and the method or process, respectively. Panel (c) shows the effect of the sampling period for each method and process (averaged over all dynamic error variances and sampling frequencies). Here, it can be seen that sampling over the entire period, rather than just the first half, resulted in larger mean MSE values for the local and global polynomial regression for all processes except the cusp‐catastrophe. This effect was (almost) absent for the GP regression, absent for the GAMs and reversed for the parametric models. Lastly, Figure 9d shows that the mean MSE generally decreased with larger sampling frequencies for each method and process (averaged over all dynamic error variances and sampling periods).

**FIGURE 10:**
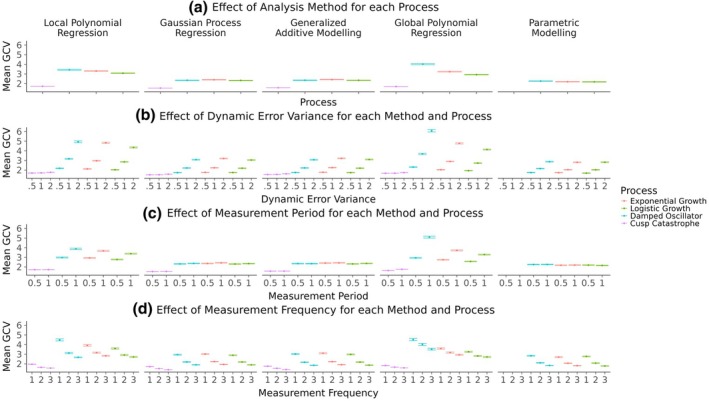
Mean GCV effects across all processes, analysis methods and simulation conditions. Panel (a) shows the effect of the analysis methods for each latent process. The other three panels show the effects of measurement period (b), measurement frequency (c) and dynamic error variance (d) for each analysis method and latent process.

Figure 10 displays the corresponding effects for the mean GCV values. Similar to the MSE results, the GAMs show a mean GCV value closest to the benchmark parametric models. However, in terms of the mean GCV, the GP regressions perform equally as well as the GAMs. The local and global polynomial regressions show considerably larger mean GCV values for all processes except the cusp catastrophe. The effects of the dynamic error variance, measurement period and frequency on the mean GCV appear to follow largely the same patterns that were observed for the mean MSE.

#### 3.7.2 Uncertainty quantification

**FIGURE 11:**
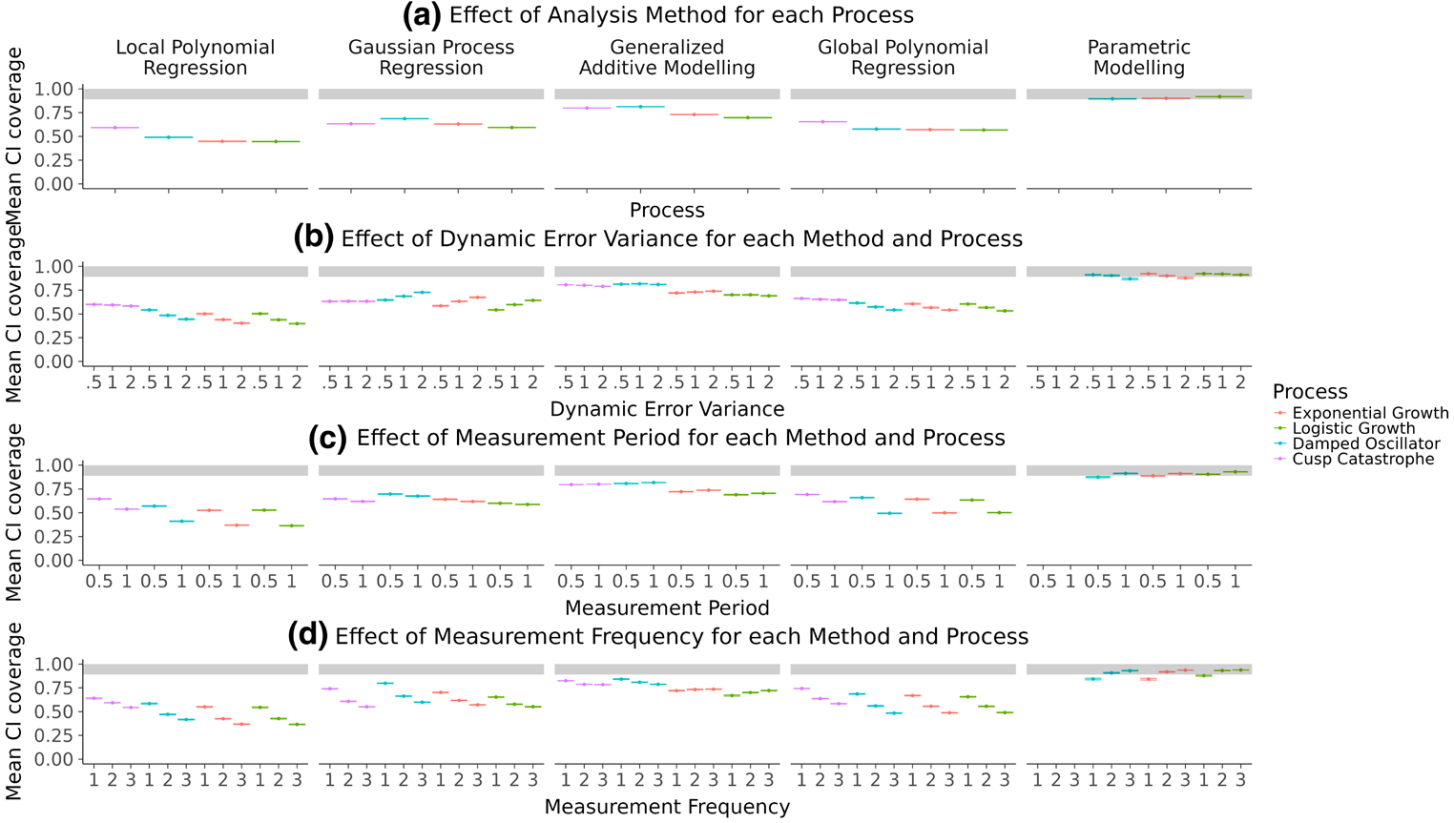
Average confidence interval coverage across all processes, analysis methods and simulation conditions. Panel (a) shows the effect of the analysis methods for each latent process. The other three panels show the effects of measurement period (b), measurement frequency (c) and dynamic error variance (d) for each analysis method and latent process.

Figure 11 shows the average confidence interval coverage proportion for the conditions described above. The grey area represents an average confidence interval coverage between 89% and 100%, which indicates no considerable deviation from the ideal 95% given the Monte Carlo error of the simulation. Only the parametric models produced some mean confidence interval coverages within this area. Among the other methods, the GAMs, produced the largest average confidence interval coverage, followed by the GP regression and then the global polynomial regression. The local polynomial regression appears to result in the smallest average confidence interval coverage.

### 3.8 Conclusion

The simulation showed that the GAMs inferred all processes with the most accuracy among the considered methods, in their default configuration, as indicated by the mean MSE, GCV and confidence interval coverage. The GAMs performed closest to the true data generating models for all outcome measures. The differences between the other three considered methods were more nuanced. We conclude that GAMs are an attractive starting point for modelling unknown non‐linear processes in ILD, especially when there is little prior theory about the functional form of the process.

Additionally, the simulation revealed that larger dynamic error variances reduced the accuracy of all methods. Therefore, addressing sources of dynamic error in practice is recommended. This can be achieved, for example, by measuring and accounting for contextual variables and other sources of external disturbances. The results also indicate that increasing the sampling frequency improved the performance of all methods, making it generally advantageous to measure processes more frequently. However, in practice, this must be weighed against considerations like participant burden and fatigue, which can negatively impact data quality. Lastly, the simulation showed that extending the sampling period enhanced the performance of the parametric models but may decrease the accuracy of LPR and polynomial regression. This reduction in accuracy for the LPR could potentially be mitigated by using extensions that allow for variable bandwidths or polynomial degrees.

## 4 AN EMPIRICAL EXAMPLE

In the following, we applied GAMs to depression data from the Leuven clinical study, which were obtained from the EMOTE database (Kalokerinos et al., n.d.). We selected the data for their heterogeneous sample, which contains momentary depression scores of participants who met the DSM criteria for mood disorders or borderline personality disorder during an intake, as well as depression scores for healthy controls. For a more thorough sample and data description, including screening protocols, see Heininga et al. (2019). The diversity in the study population makes it likely to find non‐linear processes for at least some participants.

The data set used for this application contained 77 participants in the clinical sample and 40 participants in the control sample, who were matched to the clinical sample by gender and age, resulting in a total sample size of 117.^8^ The participants completed seven days of semi‐random EMA assessments, with 10 equidistant assessments per day. During each assessment, participants responded to the item ‘How depressed do you feel at the moment?’ on a scale ranging from 0 to 100 to assess their momentary depression.^9^

The initial study procedure was approved by the KU Leuven Social and Societal Ethics Committee and the KU Leuven Medical Ethics Committee. This secondary data analysis was approved by the Ethics Review Board of the Tilburg School of Social and Behavioral Sciences (TSB RP FT16). The complete code for this analysis can be found in Appendix S1.

### 4.1 Analysis and results

**FIGURE 12:**
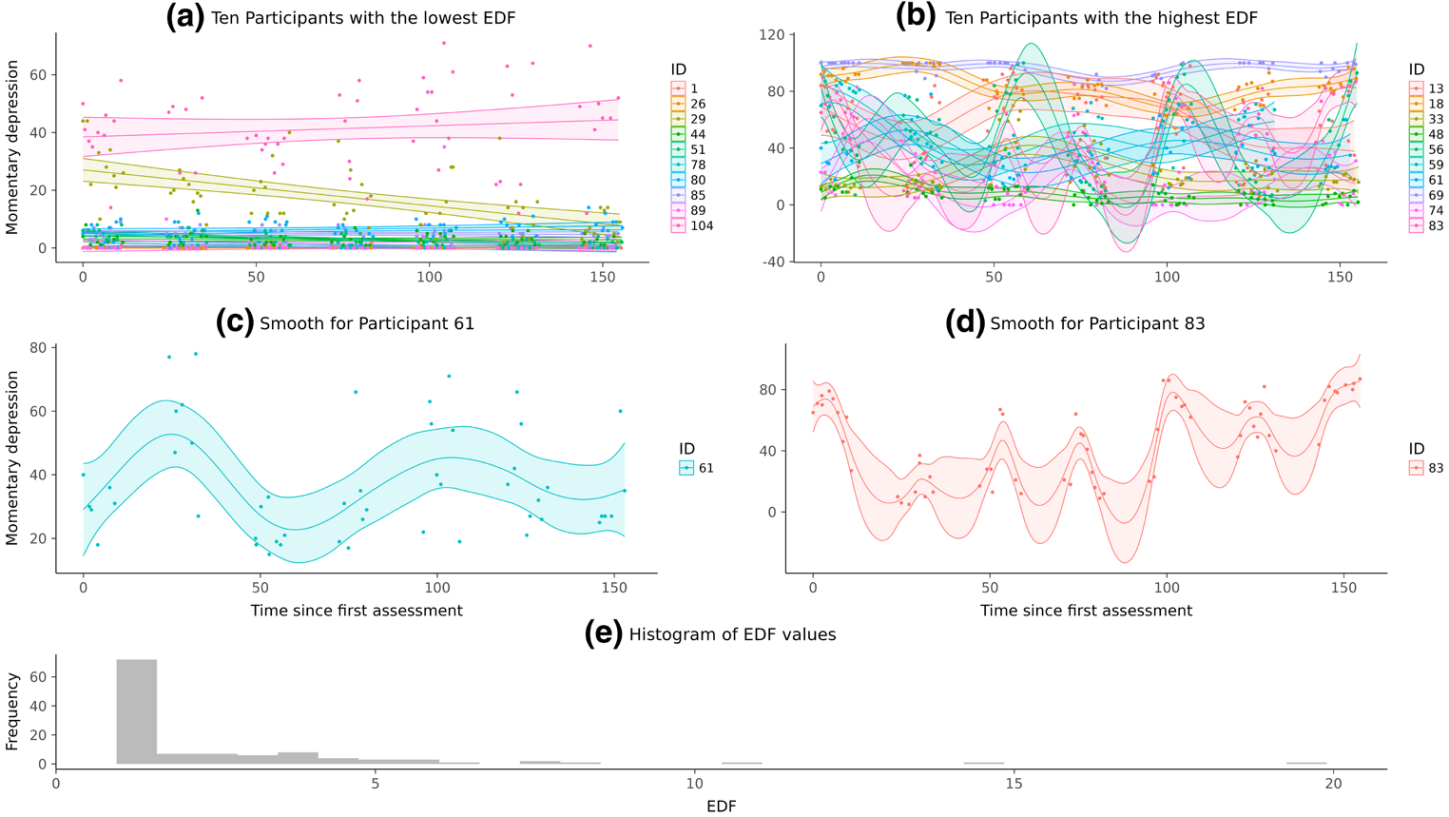
Estimated depression processes.

To maintain consistency with how the GAMs were introduced in this article and used in the simulation, we applied GAMs with a single smooth term to explore the idiographic processes underlying the data of each individual. Inspecting the estimated processes revealed a clear picture of the heterogeneity in the processes underlying the data. On the one hand, Figure 12 (a) shows the 10 estimated processes with the lowest EDF, representing the least wiggly estimated processes, which appear to be essentially linear. In fact, for 72 out of 117 participants, the processes inferred by the idiographic GAMs were effectively a linear trend (EDF < 1.001). These participants form the largest group within the sample that exhibits functionally similar processes. Due to the linearity of their estimated processes, this group may be well described by, for example, a linear mixed effects model. However, there also appear to be further structural differences between the participants in this group. For some of these participants, the linear estimates appear to be due to strong floor effects where participants repeatedly indicate depression scores close to zero. However, this explanation does not hold for all participants, as some participants also received linear estimates without displaying floor effects.

On the other hand, the remaining 45 participants had wigglier estimated processes (EDF > 1.001), as shown by the EDF distribution in Figure 12e. For these participants, the estimated processes clearly deviate from a linear trend and follow more complex trajectories. Figure 12b, for example, shows the 10 participants with the wiggliest estimated processes, indicated by the largest EDF (between 5.44 and 19.32). Notably, visual inspection of the estimated non‐linear processes did not reveal any common behaviours across the processes of different participants. With respect to the processes introduced in this article, two participants show particularly interesting trajectories. Their trajectories are shown in Figure 12c,d respectively. The process in Figure 12c closely resembles the decreasing oscillations exhibited by a damped oscillator. The process in Figure 12d shows a participant who may be switching between a low and a high depression state, with considerable oscillations within each state. A more formal analysis of the between‐person similarities and differences exhibited by the estimated idiographic processes could be achieved through, for instance, a cluster analysis. However, this is beyond the scope of this illustration.

### 4.2 Conclusion

This application demonstrates how GAMs can be used to explore potentially non‐linear idiographic processes and identify possible empirical phenomena. In these depression data, the most notable finding is that the GAMs inferred a linear trend for most participants but detected non‐linear trajectories for some individuals. For individuals with a linear trend, the results suggest the absence of dynamic errors, which would otherwise result in deviations from the linear trend. This could, for instance, indicate that these participants are not reactive to external influences. Similarly, for individuals with non‐linear trends, the observed deviations from a linear trend could also be explained by stronger dynamic errors in these individuals. In either case, the results of this application suggest considerable heterogeneity within the sample, whether in the linearity of idiographic trends or individual reactivity to external influences. This highlights the importance of using non‐parametric methods to explore idiographic processes, as a misspecified parametric model would likely obscure such qualitative differences, and insights from non‐parametric exploration can offer valuable guidance for future theories.

## 5 DISCUSSION

In this paper, we introduced three advanced semi‐ and non‐parametric regression techniques for estimating non‐linear processes in psychological ILD (Section 2). These methods address many of the limitations inherent in polynomial regression, which currently is the most common approach for modelling non‐linearity in psychology. A simulation study (Section 3) further showed that the introduced methods inferred the types of non‐linear processes that were previously found in psychological ILD more accurately than a polynomial regression. Particularly, GAMs closely approximated the underlying processes, performing almost as well as the data‐generating parametric models. Finally, we showed how GAMs can be used to explore idiographic processes and identify potential phenomena from data (Section 4). This comprehensive analysis empowers psychological researchers to accurately model non‐linear processes and to select analysis methods that align with their research goals and data characteristics.

The results obtained in the simulation aligned with almost all of our prior hypotheses. The first hypothesis that was falsified is that the cusp‐catastrophe process was inferred most accurately by all methods. We had anticipated that the smooth, continuous estimates produced by all methods would struggle to adapt to the jumps exhibited by this process. However, this effect seems to have been overshadowed by the cusp‐catastrophe's strong resilience to external perturbations. This property is highlighted in Figure 7, where dynamic errors with the same variance have been applied to all four processes. Despite the perturbations being of equal variance, the cusp‐catastrophe model appears to be the least affected and even closely resembles the unperturbed process (Figure 1). Further evidence of this can be seen in the simulation, where the effect of increasing the dynamic error variance was weakest for the cusp‐catastrophe.^10^ The second hypothesis that was in partial misalignment with the findings is that we expected the GP to perform worse for longer sampling periods (for the two growth curves and the damped oscillator). This effect was only pronounced for the confidence interval coverage and (nearly) absent for the mean MSE and GCV.

Regarding the presented simulation results, it is important to note that the observed differences in performance may be mainly caused by the specific configurations used for each method rather than the general methods themselves. The specific configurations of each method were chosen to reflect how each method is most commonly applied in practice and not to optimally infer the simulated processes. Consequently, different configurations and extensions are likely to improve the performance of the LPR and GP.^11^ For example, using a GP with a Matern class kernel, which makes less strict smoothness assumptions about the process, could be expected to improve the accuracy for the rough processes considered in this simulation. Therefore, if there is prior knowledge about the form of the process available in practice, GPs are a very interesting modelling approach, due to their flexibility and interpretable parameters. However, this likely still requires fine‐tuning the configurations of the GP to one's specific conditions. Another approach to enhance the performance of GPs when estimating processes with changing wiggliness, could be to employ a warp function that adjusts the distance between time points based on their location. This approach makes it possible to indirectly describe a time‐varying lengthscale that can adapt to the changing wiggliness of the process (Tolpin, 2019). Regarding the LPR, several optimality results have been found indicating that LPR should be at least as accurate as GAMs and GPs for processes that satisfy the smoothness assumption of the LPR (Fan et al., 1997), such as the linear processes found in the presented empirical example. However, since it is typically unknown whether a process is smooth a priori, it is unclear whether these optimality results can be generalized to other psychological processes.

Lastly, the tested polynomial regression inferred the underlying processes least accurately, highlighting the limitations of this approach. The numerical model instability of large polynomial regressions can be partially mitigated by using orthogonal polynomials, which are more difficult to interpret. Based on the results presented in this paper, there appears to be no reason to use a global polynomial regression instead of one of the other presented methods in any situation in which prior theory does not strongly suggest that the process follows a low order polynomial trajectory.

The results presented in this article are accompanied by several important limitations. First, only a limited number of processes were tested in the simulation and it is possible that the presented results do not generalize to other processes. While the theory underlying each method guarantees a good performance for processes that satisfy their respective assumptions, it is generally not known how each method performs for other processes that violate their assumptions. Second, while it is necessary to use the GCV instead of the classical leave‐one‐out cross‐validation criterion to reduce the computational load of the simulation, the GCV may inadvertently favor the GP regression and the parametric models. This is because the analytic form of the GCV implicitly requires certain parameters (such as the hyperparameters of the GP covariance function and the residual variance) to be estimated on the entire sample and then be fixed (to these values) during the cross‐validation. For the GP and the parametric models this likely improves their ability to predict the omitted data points.

Finally, the scope of this paper was restricted to univariate single‐subject data with independent normally distributed measurement errors. This somewhat idealized scenario was chosen to introduce all methods accessibly within the constraints of available software. However, this does not address many of the goals and challenges researchers face when working with ILD, which frequently contains complex measurements of several psychological variables for multiple individuals. However, there are many extensions available for the introduced methods, adapting them closer to the needs of ILD. For instance, GAMs and GPs can be naturally extended to multilevel data (Karch et al., 2020; Wood, 2006). Similarly, GP regression can be used to model different relationships between the observations and the latent process by changing their associated likelihood to a psychometric model, such as a factor model (Clark & Wells, 2023; Yu et al., 2009).

There are also many ways in which the present methods can be used to study multivariate data and structural relations between different psychological variables. The reason for this is that, even though all methods were introduced in the context of inferring non‐linear processes, they can theoretically be used to estimate a wide range of non‐linear functions. This makes it possible to infer, for example, non‐linear cross‐ and autoregressive relationships from data in discrete time (Bringmann et al., 2015; Eleftheriadis et al., 2017; Rasmussen & Williams, 2006; Wood, 2006) and non‐linear differential equation models in continuous time (Yildiz et al., 2018). These models also present the exciting possibility to combine partial parametric models with non‐linear data‐driven functions to test incomplete theories.

While these extensions and possibilities exist in theory and are individually implemented in software, most are not yet available in a form that allows researchers to flexibly and accessibly adapt these methods to the specific characteristics of ILD. Therefore, it is essential that future research unifies these methods and their extensions in software designed for the applied modelling of ILD.

## AUTHOR CONTRIBUTIONS

**Jan I. Failenschmid:** conceptualization; methodology; software; data curation; investigation; formal analysis; visualization; writing – original draft. **Leonie V. D. E. Vogelsmeier:** conceptualization; methodology; investigation; supervision; funding acquisition; project administration; writing – original draft; writing – review and editing. **Joris Mulder:** conceptualization; supervision; writing – review and editing; project administration. **Joran Jongerling:** writing – review and editing; conceptualization; investigation; funding acquisition; writing – original draft; methodology; project administration; supervision; formal analysis.

## CONFLICT OF INTEREST STATEMENT

The authors declare no conflicts of interest.

## DISCLOSURE OF ARTIFICIAL INTELLIGENCE‐GENERATED CONTENT (AIGC) TOOLS

The use of AIGC tools was limited to improving spelling, grammar and general editing.

## Supporting information


Appendix S1

## Data Availability

The simulated data and code that support the findings of this study are available in Appendix S1. The data from the Leuven Clinical Study are available upon request from the EMOTE database.

## Footnotes

^1^
Meaning that the models describing the processes do not have a random component and would result in the same trajectories if they were repeated using the same starting values.

^2^
An alternative approach to this is piecewise spline regression, which constructs a (complex) non‐linear trend by joining multiple simple piecewise functions together at specific points, called knots (e.g. connecting multiple cubic functions end‐to‐end into an overarching growth curve; Tsay & Chen, 2019). This alleviates some of the previously described issues by creating localized models. These models have less correlated parameters and are therefore less prone to model instability. However, piecewise spline regression still requires researchers to carefully select the optimal piecewise functions and knot locations, which may be done manually or in a data‐driven manner. This can be problematic in practice because, as mentioned, precise guiding theories about the functional form of most psychological processes are lacking (Tan et al., 2011). This absence of clear guidance can quickly lead to misspecified models and invalid results.

^3^
Since every polynomial term in the LPR estimates one derivative term in the corresponding Taylor series approximation (Avery, 2013).

^4^
Typically in the form of the integrated squared second derivative of the estimated process.

^5^
This procedure was implemented differently in the code or each method. One example of a code implementation can be found here: https://osf.io/ye5ca.

^6^
It is also possible to estimate the Gaussian processes used in the simulation in brms (Bürkner, 2017). However, the direct implementation in STAN, which is the Bayesian inference software underlying brms, is computationally more efficient within the context of a simulation study.

^7^
Further information regarding the proportion of models that did not converge in each condition can be found in the Appendix S1.

^8^
The original published data set contained one additional participant who was removed for this analysis since they had a depression score of zero across all assessments.

^9^
Note that this was only one of 27 items that were assessed at every measurement occasion. The other items (questions about emotions, social expectancies, emotion regulation, context and psychiatric symptoms) are, however, not relevant to this application and, therefore, not further described.

^10^
Further, rerunning the simulation with considerably smaller dynamic error variances resulted in the cusp‐catastrophe being inferred least accurately.

^11^
Especially, since the GAMs used in this simulation can be expressed as a (unconventional) GP with a linear mean function and a non‐stationary covariance function (Rasmussen & Williams, 2006; Wahba, 1978), which implies that there exists a GP configuration which performs at least as well as the GAMs.
